# Supplementary material for: The Cardiotonic Steroid Marinobufagenin Is a Predictor of Increased Left Ventricular Mass in Obesity: The African-PREDICT Study
Source: Nutrients. 2020 Oct 18;12(10):3185. doi: 10.3390/nu12103185 (PMC7603247; doi:10.3390/nu12103185)
Supplement: Supplementary file 1 [file nutrients-12-03185-s001.pdf]

Supplementary Tables

TABLE S1. Characteristics of 275 participants followed over 4.5 years compared with 927 participants from the African-PREDICT cohort.

	**Participants included in this sub study N=275**	**Participants from African-PREDICT cohort not included in analyses N=927**	**Mean difference (95% CI of diff.)**	**P**
Men, N (%)	125 (45.5)	453 (48.9)		0.32
Black, N (%)	138 (50.2)	469 (50.6)		0.91
Age (years)	25.4 ± 3.16	24.3 ± 3.06	-1.11 (-1.53; -0.70)	**<0.001**
**Anthropometric measurements**				
Height (cm)	168 ± 9.23	168 ± 9.60	0.10 (-1.19; 1.38)	0.88
Weight (kg)	73.3 ± 18.3	70.6 ± 17.0	-2.76 (-5.09; -0.43)	**0.020**
BMI (kg/m^2)	25.8 ± 5.79	24.8 ± 5.43	-0.99 (-1.74; 0.25)	**0.009**
**Frequency of obesity based on:**				
BMI ≥ 30 kg/m^2, N (%)	60 (21.8)	136 (14.7)		**0.005**
**Blood pressure**				
Clinic SBP (mmHg)	120 ± 12.4	119 ± 12.0	-0.93 (-2.56; 0.69)	0.26
Clinic DBP (mmHg)	78.9 ± 8.07	78.4 ± 7.85	-0.63 (-1.69; 0.44)	0.25
**Echocardiography**				
LVMi (g/m^2)	70.7 ± 15.7	74.0 ± 18.6	2.93 (0.66; 5.22)	**0.012**
**Urinary profile**				
24hr MBG excretion (nmol/day)	3.38 (1.12; 9.13)	3.32 (1.06; 9.30)		0.70
Estimated salt intake (g/day)[ꜝ]	7.73 (2.80; 19.4)	7.76 (2.55; 20.3)		0.92

Data presented as mean ± SD and geometric mean (5th and 95th percentiles)

[ꜝ] Estimated salt intake based on 24hr sodium excretion

BMI, body mass index; DBP, diastolic blood pressure; LVMi, left ventricular mass index; MBG, marinobufagenin

TABLE S2A. Characteristics of 215 participants with a BMI < 30kg/m² (non-obese) followed over 4.5 years.

	Baseline	Follow-up	Difference	P
Men, N (%)	101 (47.0)	101 (47.0)		
Black, N (%)	110 (51.2)	110 (51.2)		
Age (years)	25.3 ± 3.16	29.9 ± 3.23	4.57 (4.45; 4.69)	**<0.001**
**Anthropometric measurements**				
Height (m)	1.69 ± 0.09	1.69 ± 0.09	0.00 (-0.001; 0.001)	0.92
Weight (kg)	66.7 ± 12.5	71.1 ± 15.2	4.46 (3.17; 5.75)	**<0.001**
Waist circumference (cm)	76.3 ± 9.43	78.2 ± 10.4	1.88 (1.13; 2.63)	**<0.001**
BMI (kg/m²)	23.4 ± 3.32	27.8 ± 4.36	1.42 (1.10; 1.73)	**<0.001**
WHtR	0.45 ± 0.05	0.46 ± 0.06	0.01 (0.01; 0.02)	**<0.001**
**Blood pressure**				
Clinic SBP (mmHg)	119 ± 12.6	115 ± 12.6	-3.84 (-5.14; -2.55)	**<0.001**
Clinic DBP (mmHg)	78.5 ± 8.07	78.6 ± 9.18	0.13 (-0.89; 1.15)	0.81
Central SBP (mmHg)	108 ± 9.40	109 ± 10.2	0.40 (-0.67; 1.48)	0.46
Hypertension, N (%)#	27 (12.6)	29 (13.5)		0.86
**Echocardiography**				
LVMi (g/m²)	70.5 ± 15.9	76.3 ± 18.3	5.81 (3.67; 7.95)	**<0.001**
IVS_d (cm/m)	0.46 ± 0.10	0.52 ± 0.09	0.05 (0.04; 0.07)	**<0.001**
$LVID_d$ (cm/m)	2.80 ± 0.24	2.75 ± 0.23	-0.06 (-0.08; -0.03)	**<0.001**
PWT_d (cm/m)	0.48 ± 0.08	0.53 ± 0.09	0.04 (0.03; 0.06)	**<0.001**
EDVi (mL/m)	61.9 ± 12.8	58.7 ± 12.8	-3.14 (-4.48; -1.80)	**<0.001**
SVi (ml/$m^{2.04}$)	24.2 ± 5.22	22.6 ± 4.82	-1.69 (-2.30; -1.07)	**<0.001**
**Urinary profile**				
eGFR (ml/min/1.73m²)	111 ± 16.9	108 ± 16.9	-3.09 (-5.07; -1.12)	**0.002**
MBG exc. (nmol/day)	3.34 (1.12; 9.13)			
Estimated NaCl intake (g/day)	7.63 (2.80; 20.0)	7.10 (1.62; 22.8)	-0.29 (9.15)	0.60
**Biochemical profile**				
Glucose (mmol/L)	4.59 ± 0.79	4.01 ± 0.65	-0.58 (-0.71; -0.45)	**<0.001**
HDL-C (mmol/L)	1.39 ± 0.41	1.30 ± 0.34	-0.10 (-0.13; -0.06)	**<0.001**
LDL-C (mmol/L)	2.70 ± 0.90	2.56 ± 0.85	-0.14 (-0.23; -0.04)	**0.006**
C-reactive protein (mg/L)	0.80 (0.10; 6.15)	0.86 (0.14; 7.77)	0.00 (1.17)	0.27
γ-glutamyl transferase (U/L)	21.0 (8.76; 64.3)	20.1 (7.22; 62.4)	-0.37 (8.16)	0.30

Data presented as mean ± SD and geometric mean (5th and 95th percentiles)
Difference from baseline to follow up represented as mean (95% CI) for normally distributed data and median (IQR) for non-parametric data
Hypertension: Clinic SBP ≥ 140mmHg/DBP ≥ 90mmHg
BMI, body mass index; DBP, diastolic blood pressure; EDVi; end diastolic volume index; eGFR: estimated glomerular filtration rate; HDL-C: high density lipoprotein cholesterol; IVSd: interventricular septum at end-diastole; LDL-C: low density lipoprotein cholesterol; LVIDd: LV internal diameter at end-diastole; LVMi, left ventricular mass index; MBG, marinobufagenin; PWTd: posterior wall thickness at end-diastole SBP, systolic blood pressure; SVi, stroke volume index; WC, waist circumference; WHtR, waist/height ratio

TABLE S2B. Characteristics of 60 participants with a BMI ≥ 30kg/m² (obese) followed over 4.5 years.

	Baseline	Follow-up	Difference	P
Men, N (%)	24 (40.0)	24 (40.0)		
Black, N (%)	28 (46.7)	28 (46.7)		
Age (years)	25.8 ± 3.18	30.4 ± 3.15	4.68 (4.48; 4.89)	**<0.001**
**Anthropometric measurements**				
Height (m)	1.67 ± 0.09	1.67 ± 0.09	0.001 (-0.002; 0.003)	0.57
Weight (kg)	97.1 ± 15.9	102 ± 16.9	5.30 (2.90; 7.69)	**<0.001**
Waist circumference (cm)	100 ± 12.7	101 ± 12.8	0.87 (-1.35; 3.09)	0.44
BMI (kg/m²)	34.4 ± 4.34	36.3 ± 5.21	1.86 (1.00; 2.72)	**<0.001**
WHtR	0.60 ± 0.07	0.60 ± 0.07	0.005 (-0.01; 0.02)	0.42
**Blood pressure**				
Clinic SBP (mmHg)	124 ± 10.5	121 ± 12.3	-3.47 (-6.56; -0.38)	**0.029**
Clinic DBP (mmHg)	80.8 ± 7.90	81.7 ± 10.1	0.93 (-1.39; 3.26)	0.43
Central SBP (mmHg)	113 ± 8.90	116 ± 9.54	2.58 (0.17; 4.99)	**0.037**
Hypertension, N (%)[#]	12 (20.0)	13 (21.7)		1.00
**Echocardiography**				
LVMi (g/m²)	71.7 ± 15.1	83.1 ± 19.2	11.3 (6.43; 16.2)	**<0.001**
IVS_d (cm/m)	0.50 ± 0.10	0.59 ± 0.09	0.09 (0.06; 0.11)	**<0.001**
$LVID_d$ (cm/m)	2.99 ± 0.23	2.89 ± 0.21	-0.10 (-0.16; -0.04)	**0.002**
PWT_d (cm/m)	0.54 ± 0.09	0.60 ± 0.10	0.06 (0.03; 0.10)	**<0.001**
EDVi (mL/m)	71.5 ± 14.3	65.8 ± 12.0	-5.68 (-8.77; -2.59)	**0.001**
SVi (ml/$m^{2.04}$)	28.3 ± 5.41	25.1 ± 5.00	-3.24 (-4.72; -1.75)	**<0.001**
**Urinary profile**				
eGFR (ml/min/1.73m²)	111 ± 15.1	107 ± 15.2	-3.33 (-7.26; 0.59)	0.095
MBG exc. (nmol/day)	3.52 (0.93; 9.34)			
Estimated NaCl intake (g/day)	8.09 (2.57; 19.3)	7.27 (1.30; 28.0)	1.08 (12.7)	0.66
**Biochemical profile**				
Glucose (mmol/L)	4.79 ± 0.66	4.36 ± 0.54	-0.43 (-0.63; -0.24)	**<0.001**
HDL-C (mmol/L)	1.14 ± 0.27	1.09 ± 0.25	-0.05 (-0.10; 0.01)	0.087
LDL-C (mmol/L)	3.18 ± 0.89	3.01 ± 1.02	-0.17 (-0.33; -0.01)	**0.041**
C-reactive protein (mg/L)	2.80 (0.47; 60.9)	2.40 (0.23; 15.5)	0.06 (4.55)	0.51
γ-glutamyl transferase (U/L)	25.4 (8.37; 60.9)	26.8 (7.12; 138)	-1.37 (14.6)	0.73

Data presented as mean ± SD and geometric mean (5th and 95th percentiles)

Difference from baseline to follow up represented as mean (95% CI) for normally distributed data and median (IQR) for non-parametric data

[#] Hypertension: Clinic SBP ≥ 140mmHg/DBP ≥ 90mmHg

BMI, body mass index; DBP, diastolic blood pressure; EDVi; end diastolic volume index; eGFR: estimated glomerular filtration rate; HDL-C: high density lipoprotein cholesterol; IVSd: interventricular septum at end-diastole; LDL-C: low density lipoprotein cholesterol; LVIDd: LV internal diameter at end-diastole; LVMi, left ventricular mass index; MBG, marinobufagenin; PWTd: posterior wall thickness at end-diastole SBP, systolic blood pressure; SVi, stroke volume index; WC, waist circumference; WHtR, waist/height ratio

TABLE S3. Multiple regression analyses with follow-up LVMi and percentage change in LVMi as dependent variables and baseline MBG excretion as the main independent variable.

	MBG excretion (nmol/day)								
	**Normal** 18.6-24.9 kg/m^2 N=123			**Overweight** 25-29.9 kg/m^2 N=73			**Obese** BMI>30 kg/m^2 N=56		
**Dependent variable**	Adj R^2	Std. β	*P*	Adj R^2	Std. β	*P*	Adj R^2	Std. β	*P*
LVMi (g/m^2)	0.37	NS		0.44	NS		0.35	0.311	**0.007**
% Δ LVMi	0.19	NS		0.29	-0.190	0.074	0.40	0.336	**0.003**
	Sensitivity analysis additionally adjusted for estimated salt intake								
LVMi (g/m^2)	0.37	NS		0.44	NS		0.35	0.311	**0.008**
% Δ LVMi	0.19	NS		0.29	-0.190	0.074	0.40	0.337	**0.003**
	Sensitivity analysis additionally adjusted for estradiol								
LVMi (g/m^2)	0.39	NS		0.46	NS		0.47	0.305	**0.007**
% Δ LVMi	0.22	NS		0.32	-0.221	**0.035**	0.50	0.344	**0.002**

Adjusted for sex, ethnicity, age, clinic SBP, eGFR, glucose, HDL, CRP, GGT and baseline LVMi. NS refers to $P>0.05$.

TABLE S4: Backward stepwise regression model with obesity defined according to BMI, WC and WHtR criteria

	Dependent variables			
	LVMi (g/m^2)		% Δ LVMi	
**Obese; *N*=51**				
Adj R^2	0.36		0.37	
**Independent variables**	Std. β	*P*	Std. β	*P*
MBG excretion (nmol/day)	0.310	0.009	0.350	0.003
Glucose (mmol/L)	0.270	0.028	0.288	0.019
Baseline LVMi (g/m^2)	0.353	0.005	-0.570	<0.001

Adjusted for sex, ethnicity, age, clinic SBP, eGFR, glucose, HDL, CRP, GGT and baseline LVMi

*Obesity: BMI >30 kg/m^2 and WC >94cm for white men; >81.2cm for black men; >80cm for white women and >81cm for black women and WHtR >0.5

TABLE S5. Backward stepwise multiple regression analyses with follow-up LVMi and percentage change in LVMi as dependent variables, and baseline estimated NaCl intake as the main independent variable.

	Estimated NaCl intake (g/day)								
	**Normal** 18.6-24.9 kg/m^2 N=123			**Overweight** 25-29.9 kg/m^2 N=73			**Obese** BMI>30 kg/m^2 N=56		
**Dependent variables**	Adj R^2	Std. β	*P*	Adj R^2	Std. β	*P*	Adj R^2	Std. β	*P*
LVMi (g/m^2)	0.37	NS		0.44	NS		0.31	NS	
% Δ LVMi*	0.19	NS		0.27	NS		0.32	0.202	0.090

Adjusted for sex, ethnicity, age, clinic SBP, eGFR, glucose, HDL, CRP, GGT and baseline LVMi

TABLE S6: Estradiol levels of men and women in the African-PREDICT study

	Men	Women	P
**Total group**	N=122	N=140	
Estradiol (pg/ml)	37.0 (21.6; 66.7)	54.2 (6.44; 305)	**<0.001**
**Normal weight**	N=58	N=62	
Estradiol (pg/ml)	40.7 (21.3; 72.2)	59.0 (6.38; 299)	**0.019**
**Overweight**	N=35	N=39	
Estradiol (pg/ml)	32.5 (19.5; 51.2)	46.2 (6.09; 254)	0.055
**Obese**	N=23	N=31	
Estradiol (pg/ml)	34.6 (21.7; 46.1)	57.6 (15.0; 426)	**0.006**

TABLE S7: Pearson correlations between the percentage change in LVMi and estradiol in men and women

	% change LVMi	
	**Men**	**Women**
**Total group**		
Estradiol (pg/ml)	r=0.009; p=0.92	r=-0.032; p=0.71
**Normal weight**		
Estradiol (pg/ml)	r=0.183; p=0.17	r=0.236; p=0.065
**Overweight**		
Estradiol (pg/ml)	r=-0.054; p=0.76	r=-0.316; p=0.050
**Obese**		
Estradiol (pg/ml)	r=-0.313; p=0.15	r=-0.303; p=0.098
